# Pathological In Vivo Analysis of Helicobacter DNA Infection in Stomach Cells Using Carbon Nanotube Microsensor

**DOI:** 10.3390/microorganisms12122531

**Published:** 2024-12-08

**Authors:** Kyung Lee, Sihyun Jun, Yeseul Oh, Seojun Lee, Ye Jun Oh, Keum Sook Kim, Suw Young Ly

**Affiliations:** 1 Biosensor Research Institute, Seoul National University of Science & Technology, Seoul 01811, Republic of Korea; yo3252837@gmail.com (Y.O.); secienseojoon@gmail.com (S.L.); yejuno163@gmail.com (Y.J.O.); kim67121@naver.com (K.S.K.); suwyoung@snut.ac.kr (S.Y.L.); 2 University of Michigan College of Pharmacy, 428 Church St, Ann Arbor, MI 48109, USA; yshjun@med.umich.edu

**Keywords:** voltammetry, stripping, helicobacter pylori, gastric patients, carbon nanotube microsensor

## Abstract

The WHO has classified Helicobacter pylori as a group 1 carcinogen for stomach cancer since early 1994. However, despite the high prevalence of Helicobacter pylori infection, only about 3% of infected people eventually develop gastric cancer.Biomolecular detections of Helicobacter pylori(HP) were compared using specially modified sensors and fluorine immobilized on a carbon nanotube (HFCNT) electrode, which yielded sensitive results. Handheld voltammetric circuits were used for optimization. An anodic voltammogram of HP molecular oxidation was obtained at 0.0 V ± 0.1 (versus the Ag/AgCl/KCl) in a 0.1 ± 0.2 M NH_4_H_2_PO_4_ electrolyte solution. Under optimized conditions, the analytical working range was 2.98 × 10^3^–22.127 × 10^−3^ CFU/mL HP using square wave (SW) stripping voltammetry, precision of R^2^ = 0.9857 ± 0.0005 (SWSV), the detection limit approached to 2.5 × 10^2^ CFU/mL HP (S/N = 3).The developed techniques have been applied to diagnosis of early-stage HP infections using stomach tissue from healthy humans and gastric patients.

## 1. Introduction

In an in vivo disease, helicobacter pylori (HP) microaerophilic Gram-negative bacteria reside in the human stomach. HP infections are associated with gastric carcinoma [1,2,3], gastric ulcers [4], and carcinoma of the liver [5]. Since many people worldwide are infected with HP bacteria [6], diagnostic HP assay is important in evaluating early-stage infections. Existing analytical discriminations have been dependent on DNA-amplified polymerase chain reaction (PCR), biosensing enzyme reaction, and other detection systems, such as multiplex polymerase chain reaction (PCR) [7], the second-generation ELISA (Cobas Core, anti-*H. pylori* EIA Roche, Basle, Switzerland) [8], enzyme-linked immunosorbent assay [9], radio active polymerase chain reaction [10], analysis of *Helicobacter pylori* infection using nanotube structure with a large surface area [11], a wide range of potential applications [12], and an electro catalytic reaction (–[C*_x_*–(H–F)*n*]*^n^*^+^M^0^↔M^+^) [13,14,15], making it applicable for biological recognition and HP diagnosis.

And, these technologies are time-consuming and complex. They have the dis advantage of having a high detection limit, and CNT materials can be useable for the human body and can be directly diagnosed within the body muscles for in vivo or in vitro experiments. Also CNTs produce acid-based sensitive electron transfer. Additionally, the electron speed is fast and it is not toxic to the human body. It amplifies the redox reaction of viral or cellular ions. Also, CNT atoms react sensitively to electrons. When using a mixture of bismuth and carbon nanotubes for this catalytic effect, sensitive detection limits can be reached [16]. Carbon nanotubes (CNTs) coupled with bismuth have a very large surface area. They are also electrically conductive. The physical properties of CNTs vary depending on the number of bonds of carbon in amouse model [17] and PCR double-strain DNA-binding method [18]. In contrast, PCR methods are dependent on chromatographic separation, electrophoresis isolation, and other detection systems. Recently, electrochemical stripping accumulation techniques have been applied to diagnostic bioassay, which is simple, fast [19], and capable of sensitive signal amplification. Specialized modified working electrode techniques have also been widely applied to clinical detections, such as infrared photo diode electrode [20], the carbon nanotube paste electrode [21,22], the mercury-modified carbon electrode [23,24], and other sensors. Nevertheless, electrochemical HP assays are rarely examined, including electrochemical (3,4-DHS) and (2,5-DHS) probes [25], an amperometricimmunereactor with rotation incorporated into an FI analytical system [26], and a screen-printed immunesensor [27]. These methods use specific properties that are still complicated and unattainable for an in vivo direct assay. In this study, a novel fluorine doped on a carbon nanotube (CNT) sensor [13] was used for HP recognition. Its carbon atoms formed the wall. A single-wall nanotube is a tube with one wall made of carbon atoms. It has excellent electrical and thermal conductivity. Therefore, it is suitable as a biological ion diagnostic material [28]. However, double-wall nanotubes with two walls have excellent electrical conductivity and mechanical properties. So, a multi-wall nanotube is a tube with multiple walls made of carbon atoms. It has more superior mechanical properties than electrical and thermal properties. It is easy to manufacture and has a wide range of applications. As for mechanical properties, it is a material with strong tensile strength and elastic modulus. This is due to the sp^2^ covalent bonds formed between carbon atoms.Multi-walled carbon nanotubes have a tensile strength of 63 to 100 (GPa: gigapascals) or more. In single-wall experiments, carbon nanotubes exhibit very high tensile modulus in the range of 640 GPa to 1 TPa. They also have a high tensile strength of 150 to 180 GPa. Therefore, the lifespan of the ion diagnostic sensor is extended [29]. However, the mechanical properties of carbon nanotubes weaken under compression conditions. Due to the high aspect ratio of CNTs, their ductility increases when subjected to compressive, torsional, or bending stresses. Furthermore, it has a bending nature. The Young’s modulus of CNTs in the radial direction is measured in GPa. Therefore, carbon nanotubes become very soft and linear in the radial direction. Also, in textile applications, specific strength is a measure of the mechanical effects of the fiber. Specific strength of the material (force per unit area at breakage) is divided by density. SI units are expressed as Pa·m^3^/kg or N·m/kg. Biotic fibers with low density show high specific strength. The density of carbon nanotubes is 1.3 to 1.4 g/cm^3^. However, the density of carbon steel is 7.85 g/cm. The specific strength of carbon steel is 154 kN·m·kg^−1^. The specific strength of carbon nanotubes is 48,000 kN·m·kg^−1^. Therefore, CNTs exhibit high specific strength, and CNTs show the best specific strength among materials with known specific gravity. The mechanical properties of carbon nanotubes are stronger than iron and aluminum and are lighter than aluminum (rho_Al = 2.0 g/cm^3^), the lightest metal among metals. Therefore, it is convenient to process and can be applied to diagnostic sensors. Carbon fiber has the disadvantage that only 1% of its molecular structure is modified. However, carbon nanotubes can withstand even 15% deformation. Carbon nanotubes can be used in a wider range of human body materials than steel, diamond, copper, and fiber. Carbon nanotubes exhibit metallic or semiconducting properties depending on the chirality of the coiled axis. Therefore, a diagnostic detection circuit can be configured. Metallic carbon nanotubes transmit electrons in the longitudinal direction without being scattered. A single-walled carbon nanotube exhibits large acoustic phonons. It has a high thermal conductivity of 6000 W/(m·K). When electricity is passed through carbon nanotubes, they emit light that is more than 100 times more efficient than LEDs (light-emitting diodes). Therefore, they can be used in photoelectric sensors. The thermal conductivity of carbon nanotubes is the same as that of diamond, which is the highest in nature. Therefore, thermoelectric sensor analysis can be used. Tensile strength exceeds diamond. Carbon nanotubes have the same level of electrical conductivity as copper. Therefore, it is possible to synthesize it with conductive resin. [30] For the above reasons, it can be used as a virus diagnostic sensor material [31]. These results can be applied to the following fields: cell diagnosis, virus identification, DNA diagnosis, wearable navigation control, unmanned self-diagnosis treatment medicine, AI-linked control, etc. [32]. It is also can be useable for synthesizing various nanoparticles for different applications for in vivo and in vitro experiments [33,34].

## 2. Materials and Method

### 2.1. Technical Systems

Voltammetric measurements were carried out using the second version of the Bioelectronics-2 system from the authors’ institution (biosensor research institute in seoul national university of science & technology). The electric circuitry had very small computerized systems, a 3.0 V potential range, a 2 mA current range, and a 10 pA measuring current. It was as compact as a cellular phone, measuring 3″ × 2″ × 1″. It used a rechargeable battery with a USB power source and was capable of USB port data telecommunication with a PC. (We developed and used the GVA 2D software control program). It can be used for bioassay, microorganism recognition, and sensor techniques for individual and laboratory conditions.

### 2.2. Sensor Preparation

The fluorine immobilized on a carbon nanotube (HFCNT) working sensor was prepared with a mixing paste of 40% carbon nanotube powder (Nanotech Co., Ltd., Asan, Choongnam, Republic of Korea, 330–816), 40% HF (sigma standard, concentrated solution), and 20% mineral oil. The mixture was homogenized in a mortar for 30 min. The paste was inserted into a 5 cmglass (parafilm coated) needle-type capillary tube that was 1.5 mm in diameter, and a copper wire that was 0.5 mm in diameter was connected to the electrochemical measurement system. The DNACNT (double-stranded DNA and carbon nanotube powder mixed paste) was prepared with the same method, using 40% DNA (double-stranded and prepared from calf thymus sigma, St. Louis, MO, USA), 40% carbon nanotube graphite powder, and 20% mineral oil. The HGCNT (metal mercury and carbon nanotube mixed paste) was made using 40% Hg (1000 mg/L mercury stranded from sigma, St. Louis, MO, USA), 40% carbon nanotube graphite powder, and 20% mineral oil. The HFCNT (conc fluorine and carbon nanotube mixed paste) was made using 40% Hf (cocn–Hf solution stranded from sigma), 40% carbon nanotube graphite powder, and 20% mineral oil. Here, the Hf, DNA, and Hg were immobilized on a carbon nanotube paste surface using a cyclic scan with a −2.0 V initial potential, a 2.0 V switching potential, and a 0.5 V scan rate for 20 repetitions in a 0.1 M NH_4_H_2_PO_4_ electrolyte solution. The three sensors were rinsed with water to remove any regent solution residue, and then dried using an air gun. The three modified CNT sensors were subsequently transferred to a cell that contained 0.1 M NH_4_H_2_PO_4_, and their SW stripping voltammograms were recorded. The graphite pencil (PE) working electrode was prepared with 5H or 2B pencil lead (2 mm in diameter) (Table 1). The reference electrode used was Ag/AgCl, and the auxiliary electrode, a 0.2mm-diameter platinum wire. A three-electrode cell was used to monitor the voltammetric signal.

## 3. Results

### 3.1. Voltammetric Procedure and the Electron Microscope

All the analytical solutions were used in 18 M ohm cm^−1^ double-distilled water. Analytical grades of a 0.1 M NH_4_H_2_PO_4_ electrolyte solution with a pH level of 4.75 and an experimental reagent from Aldrich Chemical Co. (St. Louis, MO, USA) were prepared. The voltammograms were measured in dissolved oxygen, and each measurement did not require an electrode cleaning time. The phosphoric acid solution was found to be the most suitable medium. The common parameters for the CV were a scan rate of 500 mVs^−1^, an initial potential of +2.0 V, and a switching potential of −2.0 V. The stripping voltammograms were performed using Figure 3A parameters.

The HP-cultured structure was scanned with a field-emission-scanning electron microscope (FE-SEM, JSM-6700F JEOL, Ltd., Tokyo, Japan).

### 3.2. HP Culture and Tissue Collection

The HP culture was grown at 37 °C on Columbia agar that contained 7% horse blood. The plates were incubated from 72 to 140 h in a circulate under microaerophilic conditions of 5% CO_2_, 4% O_2_, 86% N_2_, and 5% H_2_ using gas packs. The HP was washed in phosphate-buffered saline. The suspension was acidified with concentrated hydrochloric acid. It was disrupted with an ultrasonic probe at mid-power under ice cooling. Gastric tissues (0.25 g) were collected from 12 patients and 12 healthy persons, including 4 males and 20 females, ages 30 to 55. Tissues were obtained between 9 am and 5 pm at the general hospital where one of the authors worked. A liquor of gastric tissue was placed in a glass vial that contained disinfected 5 mL saline and was stored at −10°C. This was allowed to thaw at room temperature and was used directly.

The PCR DNA was isolated from the 100mLtissue solution using a Core-OneTMGenomic DNA isolation kit (Thermo Scientific™, Waltham, MA, USA), with Cat. No. TD-100 manufacturer’s guide. This was used for the proteinase K-lysis method. The DNA was eluted under a 10 mm Tris-HCl buffer and at pH 8.5 and was used for diagnostic analysis.

## 4. Results and Discussion

### 4.1. Electrode and Voltammetric Concentration Effects

**Figure 1:**
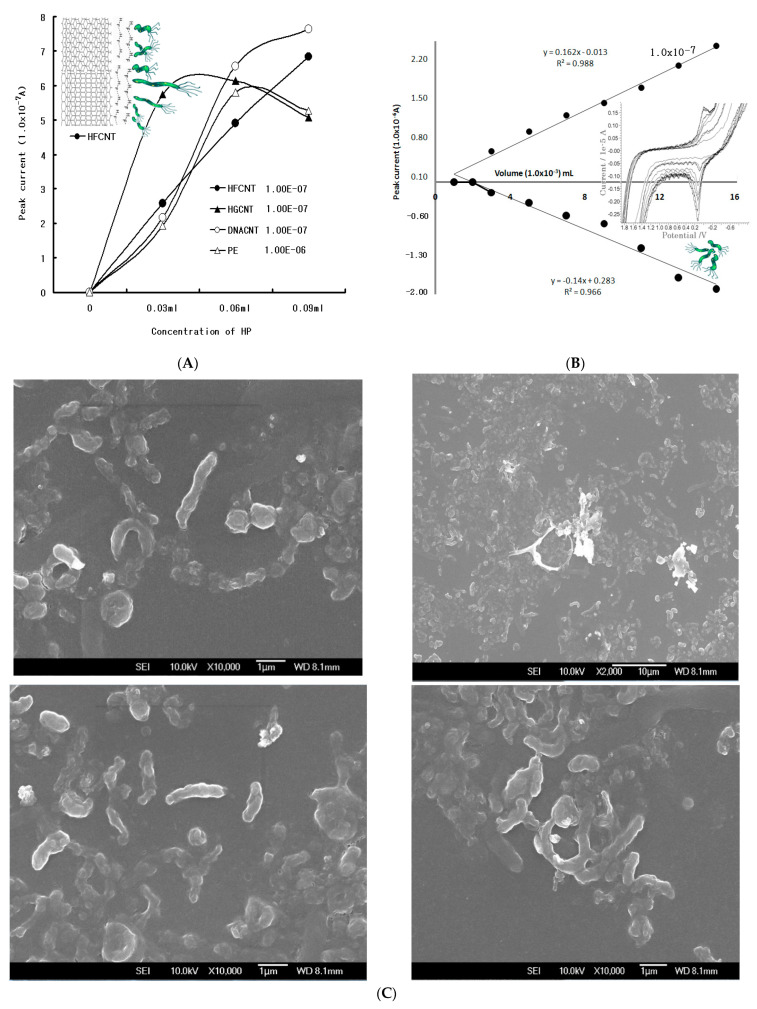
(**A**) Comparisons of the PE, HFCNT, HGCNT, and DNACNT in 0.03, 0.06, and 0.09 mL (2.98 × 10^3^ CFU/mL) HP concentrations using optimum SW anodic conditions with optimum parameters. (**B**) Cyclic voltammetry from 0.003 to 0.024 mL HP concentration via HFPE in a 0.1 M ammonium phosphate solution (pH 4.5) with optimum parameters, and theDNA sequence (5′-ATGGAAATACAACAAACACAC-3′ and 3′-CTGCTTGAATGCGCCAAAC-5′ for vacAs1/s2 while for vacAm1 and vacAm2 the primers were 5′-GTCAAAATGCGGTCATGG-3′ and 3′-CCATTGGTACCTGTAGAAAC-5′ and 5GGAGCCCCAGGAAACATTG-3′ and 3′-CATAACTAGCGCCTTGCAC-5′, respectively). (**C**) FE-SEM scanning of the cultivated HP cells showing a 10.0 kV accelerated voltage with 10,000 times magnification of a secondary electron image and width of 8.1 mm.

Since electrode properties depend on their chemical structure, common and specialized sensors were used to compare the PE, HFCNT, HGCNT, and DNACNT. Figure 1A shows a 0–0.09 mL HP concentration in SW anodic stripping voltammetry using three electrodes at optimum parameters. A 0.0 V anodic peak potential was obtained in all the sensors. While the HGCNT was sensitive and its peak width was sharp, its linear working range was very short. The DNACNT and PE were not sufficient. Since the HFCNT was linear and its peak width was sensitive, it can be used in an HP assay. Thus, the CV voltammograms determined the analytical anodic and cathodic peak potentials with an HP concentration with the addition of eight points.

Figure 1B shows low voltammograms of the 0.003–0.024 mL concentrations, but the oxidation of the 0.0 V peak currents increased from 0.183 × 10^−6^ A to 2.06 × 10^−6^ A, whereas the reduction currents varied from 0.554 × 10^−7^ A to 2.37 × 10^−7^ A. The reduction peak was poor, the oxidation was more hole-sensitive than the reduction current, and the peak width was sharp. Thus, more sensitive peak signals were detected via SW stripping optimization. Figure 1C shows the scanning electron micrograph images of the HP cells. The white images are the concentrated cell surfaces, which were used for the voltammetric standard solutions. Table 2 appears as a straight lines of least squares, however, the other electrode appeared in the form of short curves.

### 4.2. SW Parameters and Statistics

**Figure 2:**
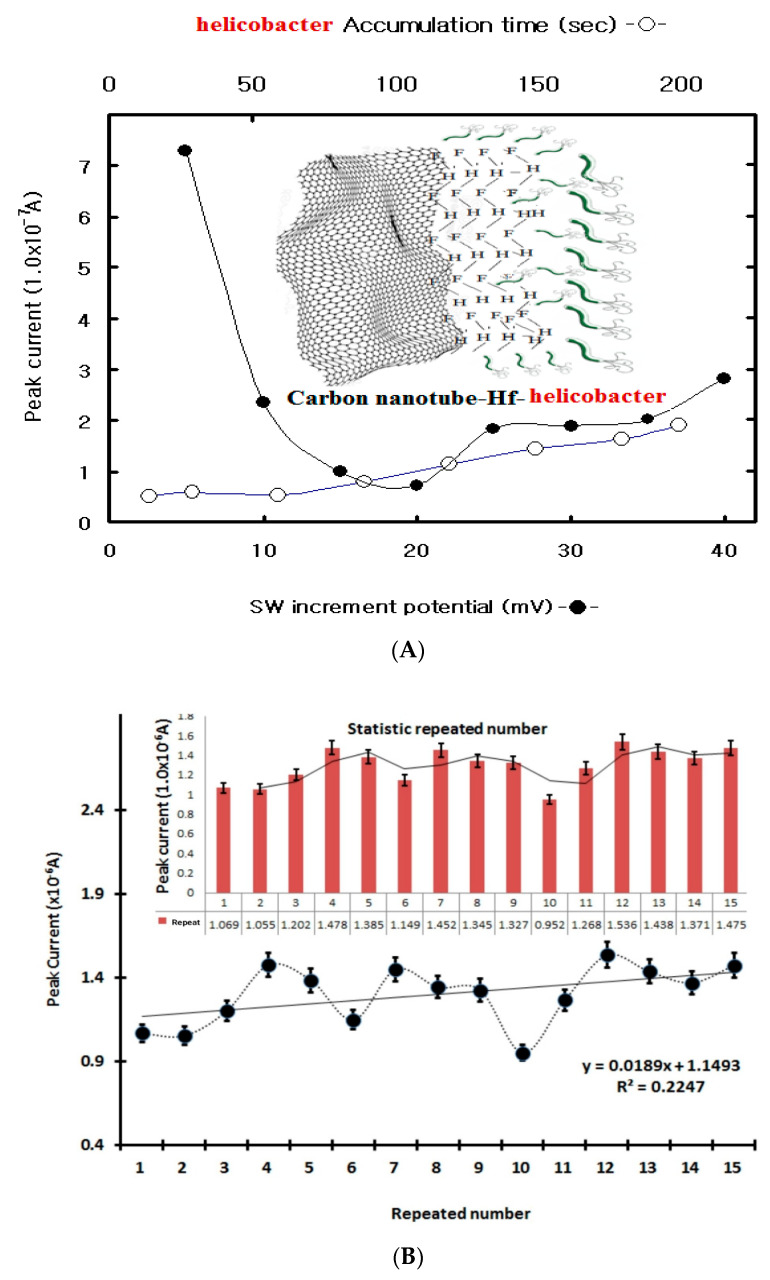
(**A**) SWSV accumulation time variations from 15 to 200 s and incremental potential variations from 5 to 40 mV with a 0.01 mL HP concentration in a 4.6-pH 0.1 M NH_4_H_2_PO_4_ electrolyte solution. (**B**) Electrode stability in the (**A**) solution for 15 repetitions with the optimum SW conditions in Figure 3A.

In SW stripping voltammetry, time variation influences signal amplification. In this study, the SW stripping accumulation time was first examined using eight-point variations. The results are shown in Figure 2A. The peak current increased from 0.53 × 10^−^^7^ to 1.90 × 10^−^^7^, but the peak width was wide and sensitive at 200 s. Thus, the accumulation time was fixed at 200 s.

Under these conditions, the SW incremental potentials were examined using eight-point variations. They decreased quickly from 7.93 × 10^−7^ A to 0.459 × 10^−7^ A; the peak width was broad and expanded; and the current was high at 5 mV and then decreased. Thus, the accumulation time was 200 s, and the incremental potential was fixed at 5 mV. Under these conditions, other parameters were examined using the initial potential, the SW frequency, and the amplitude variation. The final results were −1.0 V initial potential, a 20 Hz SW frequency, a 50 mV amplitude, a 200 s accumulation time, a 5 mV incremental potential, and a 0.1 M NH_4_H_2_PO_4_ (4.7 pH) strength. Under these conditions, electrode stability was examined with 0.01 mL HP added and a 100s accumulation time. The first five points were varied from 0.41 × 10^−6^ A to 0.92 × 10^−6^ A then increased (not shown here) before they were linearized and stabilized. The errors are shown in Figure 2B. The minimum current was 0.927 × 10^−6^ A and the maximum current was 1.594 × 10^−6^ A. The static relative standard deviation was R^2^ = 0.08376, and then usable working ranges were examined using SWSV. Table 3 shows the counts in statistical plot in Figure 2B. The error range of fifteen repeated spike measurements under the same concentration conditions is shown in the table.

### 4.3. Working Range and Application in Patients’ Stomach Tissue

**Figure 3:**
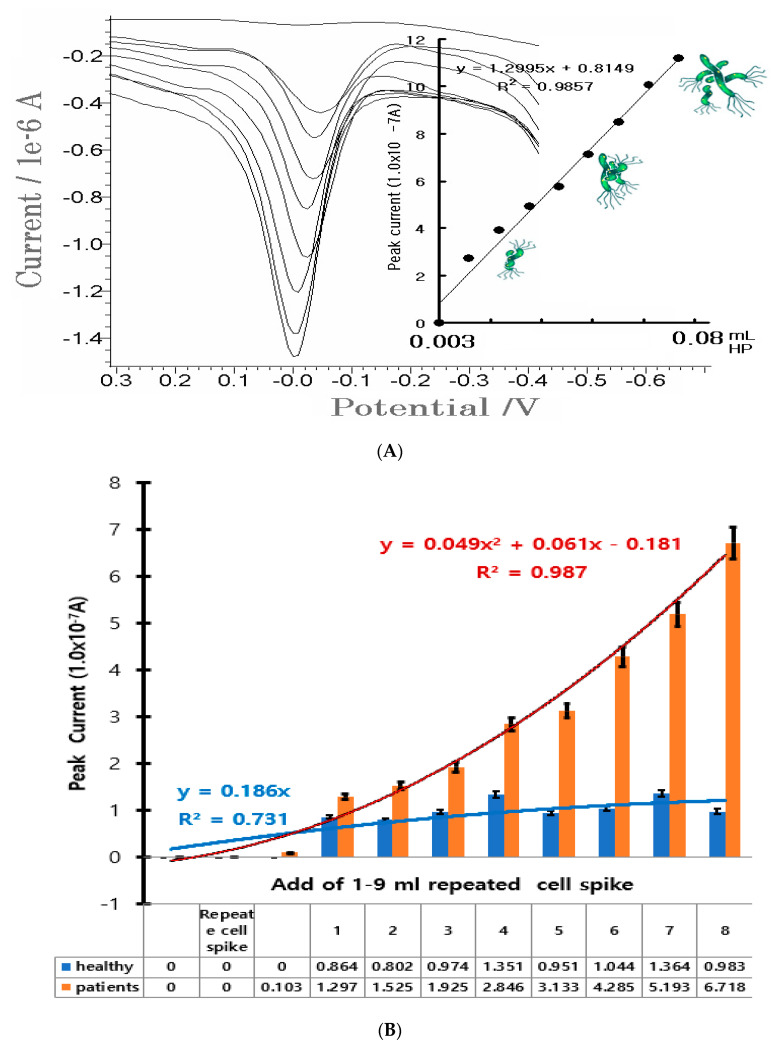

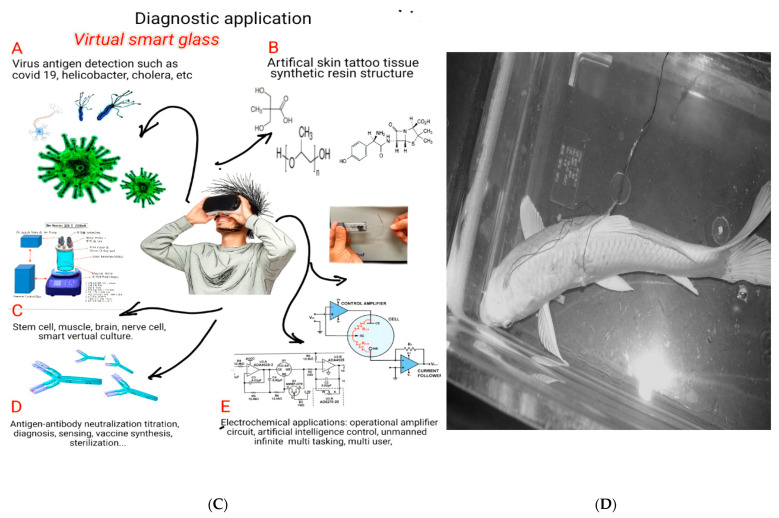
(**A**) SWSV anodic 0.003–0.08 mL HP (5.613 × 10^−3^–3.92 × 10^−4^ CFU/mL HP) added to a 0.1 M NH_4_H_2_PO_4_ solution. SW stripping: 50 mV amplitude, 20 Hz frequency, −1.0 V accumulation potential, and 200s accumulation time were used with a pH of 4.75. (**B**) Addition of 1–9 mL of diluted stomach tissue from healthy patients via SWSV. (**C**) Simulated illustration being applied, shows real time diagnosis of Helicobacter pylori virus living in the stomach, skin-coated tattoo sensor and diagnostic circuit, pain transmission neurodiagnostic network, and 3-electrode systems of amplified operational voltammetricnetwork.(**D**): Food fish. Real-time diagnostic experiment with in vivo sensor insertion.

Using optimum parameters, the linear working ranges were sought with the HFCNT set in optimum SW voltammetry. Figure 3A shows a variation from 0.01 to 1.0 × 10^−^^9^ mL HP. The first curve refers to the electrolyte blank solution. It was simple and did not manifest any signal, whereas the other voltammograms sharply increased from 2.2 × 10^−^^7^ A to 11.6 × 10^−^^7^ A with nine points, and linear equations were obtained at Δx/Δy = 1.2995x + 0.8149.

The slope coefficient: was sensitive and precision was R^2^ = 0.9857 ± 0.0005. Detection time was faster and more sensitive than that of other common analytical methods. Low concentration curves for detection limits are not attached here. However, low concentration experiments were repeated on micro spiking, and the blank electrolyte was repeated three times under optimal experimental para conditions.

The results were reached with a lower detection limit of 2.5 ± 0.5 × 10^−2^ CFU/mL HP (S/N = 3), according to the IUPAC recommendation (3σ).

Also, the selective properties of similar inorganic and organic ions were not tested. The reason is that the reaction between antibodies and antigens does not produce an error of more than 5%. Moreover, the SW peak current was more sensitive than the CV peak. These indicate feasible use in the recognition of HP infection and gastric carcinoma. To validate, an analytical application was carried out on certain patients using healthy stomach tissue. About 0.25 g of human stomach cells were extracted from a healthy patient and flushed with water and diluted in one drop of 0.1 M HCl. The sample was then further diluted in 10 mL distilled water. The 0.1 mL solutions were directly used in the SW stripping voltammetry. At first, the two additions did not exhibit any peak current. Thus, continued additions were performed with 1–9 mL concentrations. Figure 3B shows the results from the healthy (black) and diseased (white) stomach tissue. The “healthy” curve had no signal, and only noise was obtained at the range of 0.2 × 10^−7^–0.95 × 10^−7^ A. The “unhealthy” curve increased, however, from 0.5 × 10^−7^ to 8.8 × 10^−7^ A, and was 10 times more sensitive than the healthy tissues. These were subsequently used for HP analysis. Thus, the developed techniques can be applied in the diagnosis of HP infection. Expanded statistic applications were performed with 10 patients and 10 healthy persons, and a recognition rate of 70–80% was attained. The proposed methods can be used in patient diagnosis and in vivo direct assay. Figure 3C shows such a simulation diagram being applied and shows real-time diagnosis of a Helicobacter pylori virus living in the stomach of a human organism.In a counterclockwise direction, it describes the following. 1:The HP virus real-time diagnostic application functionin the body.2: The nerve signal transmission real-time diagnostic control application function. 3:The microchip, electrochemical diagnostic amplification circuit.4: Human-skin-coated tattoo sensors of 3-electrode systems of working, counter, and reference films. Table 4 showsthe results data in the statistical diagram with the repeated input of real normal and patient samples in Figure 3A,B. We applied experiments for application to the human body. Figure 3Dis an experimental picture of edible fish instead of a human experiment. The three electrodes, working, counter and reference, were made of a muscle-embedded type of material. A 95% copper wire, 0.5 mm in diameter and 10 mm long, was used. The working electrode was coated and synthesized with CNT + conductive resin mixture paste. Paste was repeatedly coated on the 95% Cu wire three times and then dried. The counter and reference electrodes were made of pure copper and of the same size. The counter and reference electrodes were inserted into the 10 mm muscle on the left and right wings and in the right wing. After the insertion, the open wound was sutured with a wood adhesive. The working electrode was inserted into the head of the center of the brain at a depth of 10 mm. Which cable was connected to the back muscle with a surgical thread. And then was connected to the voltammetry system. The real-time in vivo experiment was repeatedly measured under conditions and concentration experiments in the same way. Similar results to the electrolyte experiment were obtained. These results reached a level that can be applied directly to the human body. However, data were not attached to this study. The picture is a real-time in vivo and in vitro diagnostic picture.Therefore, the same experiment for the human body could be replaced.

## 5. Conclusions

Biomolecules of HP were searched using handheld voltammetric circuits. Their analytical sensitivities were compared using a common and novel HFCNT. The novel HFCNT was found to be more sensitive than common sensors. Optimum conditions comprising a 200s accumulation time, a −1.0 V SW initial potential, and a 4.75 ± 0.05-pH electrolyte strength were attained. The optimum conditions reached a lower detection limit of 2.5 ± 0.5 × 10^−2^ CFU/mL HP (S/N = 3), according to the IUPAC recommendation (3σ), which is a more sensitive detection limit and faster time than other reports of minimum of 10,000 CFU/mL [35], detection of 0.25 fg/µL of the *H. pylor* DNA [36], and minimum inhibitory concentration >1.0 μg/mL [37]. These results indicate that the sensor systems developed can be used in the detection of early-stage HP infection in the stomach tissue of human organic cells and in vivo body fluid with a catheter-type sensor. They can also be applicable in any other field that requires a real-time, in-organ direct assay.

## Figures and Tables

**Table 1:** Detailed material summary of the sensor synthesis circuit diagram.

Sensor Type	(HFCNT) The Fluorine Immobilized on aCarbon Nanotube	(DNACNT) Double-Stranded DNA and Carbon Nanotube Powder Mixed Paste	(HGCNT) Metal Mercury and Carbon Nanotube Mixed Paste	(PE) The Graphite Pencil
Synthetic material	Mixing paste of 40% carbon nanotube powder (Nanotech Co., Ltd., Choongnam, Republic of Korea, 330–816), 40% HF (sigma standard, concentrated solution), and 20% mineral oil.	In total, 40% DNA (double-stranded and prepared from calf thymus sigma), 40% carbon nanotube graphite powder, and 20% mineral oil.	In total, 40% Hg (1000 mg/L mercury stranded from sigma), 40% carbon nanotube graphite powder, and 20% mineral oil.	Prepared with 5H or 2B pencil lead (2 mm in diameter).
Sensor sensitivity/ concentration lange	High sensitivity <10 μg ~/L	High sensitivity <10 μg ~/L	High sensitivity <10 μg ~/L	Low sensitivity <100 μg ~/L

**Table 2:** HFCNT concentration effects at 100 s accumulation time in same electrolyte conditions.

Sw Stripping Voltammetry	HFCNT		100 s Accumulation Time
0.003 mL^−1^, Repeated Spike	*x*-Axis HP Concentration Number	CFUs/mL	Peak Current (10^−7^)
0	0	0	0
1	5.29	29,694.53157	2.728
2	7.639	42,880.25079	3.899
3	9.695	54,421.26344	4.924
4	11.369	63,817.98288	5.759
5	14.089	79,086.24864	7.115
6	16.81	94,360.12773	8.472
7	19.993	11,2227.3667	10.059
8	22.202	12,4627.2193	11.16

**Table 3:** Data counts in statistical plot in Figure 2B.

Sw Stripping Voltammetry	Accumulation Time 100 s	
0.01 mL. HP Spike	10X^-6^ Amper Current	
1	1.069	Average coefficient: 1.3412
2	1.055	Standard deviation: 0.08376
3	1.202	
4	1.478	RSD = 0.062452
5	1.385	Relative standard deviation: RSD = s/X averge
6	1.149	Coefficientof variation: CV = (s/x) × 100%
7	1.452	
8	1.345	
9	1.327	
10	0.952	
11	1.268	
12	1.536	
13	1.438	
14	1.371	
15	1.475	

**Table 4:** Number of data in the statistical diagram with repeated input of normal and patient sample spike in Figure 3A,B.

	Healthy	Patients	
	100 s	100 s	
Repeated Cell Spike	10^−7^ Peak Current	10^−7^ Peak Current	
	0	0.103	healthy: Y = 0.186X
1	0.864	1.297	
2	0.802	1.525	patients: Y = 0.049X^2^ + 0.061X − 0.181
3	0.974	1.925	R^2^ = 0.987
4	1.351	2.846	
5	0.951	3.133	
6	1.044	4.285	
7	1.364	5.193	
8	0.983	6.718	

## Data Availability

All data generated or analyzed during this study are included in this published article.

## Abbreviations

HP: Helicobacter pylori; HFCNT: fluorine immobilized on a carbon nanotube; SW: square; SWSV: stripping voltammetry; PCR: polymerase chain reaction; PE: graphite pencil;HFCNT: fluorine immobilized on a carbon nanotube; DNACNT: double-stranded DNA; HGCNT: metal mercury and carbon nanotube mixed paste.
